# Milestones in Mandibular Bone Tissue Engineering: A Systematic Review of Large Animal Models and Critical-Sized Defects

**DOI:** 10.3390/jcm14082717

**Published:** 2025-04-15

**Authors:** Yannick M. Sillmann, Pascal Eber, Elizabeth Orbeta, Frank Wilde, Andrew J. Gross, Fernando P. S. Guastaldi

**Affiliations:** 1 Department of Oral and Maxillofacial Surgery, Massachusetts General Hospital, Harvard School of Dental Medicine, Boston, MA 02115, USA; yannick.sillmann@uni-ulm.de (Y.M.S.); peber@mgh.harvard.edu (P.E.); 2 Department of Oral and Plastic Maxillofacial Surgery, University Hospital Ulm, 89081 Ulm, Germany; frank.wilde@uni-ulm.de; 3 College of Dental Medicine, Western University, Pomona, CA 91766, USA; elizabeth.orbeta@westernu.edu; 4 Department of Oral and Plastic Maxillofacial Surgery, Military Hospital Ulm (Academic Hospital of the University of Ulm), 89081 Ulm, Germany; 5 Division of Oral and Maxillofacial Surgery, University of Utah, Salt Lake City, UT 84112, USA; andrew.gross@hsc.utah.edu

**Keywords:** mandibular reconstruction, bone tissue engineering (BTE), large animal models, critical-sized defects, scaffold

## Abstract

**Background/Objectives:** Mandibular reconstruction following trauma or oncologic resection is crucial for restoring function and aesthetics. While autologous bone grafting remains the gold standard, it presents challenges such as donor site morbidity and graft availability. Bone tissue engineering (BTE) offers an innovative alternative, integrating scaffolds, osteogenic cells, and bioactive factors to regenerate functional bone. This systematic review evaluates BTE strategies for mandibular reconstruction, focusing on critical-sized defects in large animal models and their translational potential for clinical applications. **Methods:** A systematic review was performed following PRISMA guidelines. Eligible studies involved large animal models and critical-sized mandibular defects treated with at least two BTE components (scaffold, osteogenic cells, or growth factors). Quality and bias assessments were conducted using ARRIVE guidelines and SYRCLE tools. **Results:** Of the 6088 studies screened, 27 met the inclusion criteria, focusing on critical-sized mandibular defects in large animal models such as pigs, sheep, and dogs. Common scaffolds included β-tricalcium phosphate (β-TCP), poly-lactic-co-glycolic acid (PLGA), and polycaprolactone (PCL), frequently combined with bone marrow-derived mesenchymal stem cells (BMSCs) and growth factors like recombinant human bone morphogenetic protein-2 (rhBMP-2). Preclinical outcomes demonstrated effective bone regeneration, vascularization, and biomechanical restoration. Advanced strategies, including in vivo bioreactors and 3D-printed scaffolds, further enhanced regeneration. However, challenges such as incomplete scaffold degradation, hypoxic conditions within constructs, and variability in growth factor efficacy and dose optimization were observed, emphasizing the need for further refinement to ensure consistent outcomes. **Conclusions:** BTE shows promise in mandibular reconstruction, achieving bone regeneration and functional restoration in preclinical models of critical-sized defects. However, challenges such as scaffold optimization, vascularization enhancement, and protocol standardization require further investigation to facilitate clinical translation. These findings emphasize the need for refinement to achieve consistent, scalable outcomes for clinical use.

## 1. Introduction

Mandibular resection, often necessitated by oncologic disease or trauma, leads to substantial functional and aesthetic impairments, severely affecting mastication, deglutition, speech, and in severe cases, even airway patency. These deficits impact essential functions such as chewing, swallowing, and speaking, and in severe cases, can lead to critical complications like airway collapse, significantly diminishing social interactions and consequently the patient’s quality of life [1,2]. Reconstructing the mandible is essential for patient rehabilitation and restoring both form and function [2].

Despite bone tissue’s intrinsic ability to regenerate, the challenge of repairing large mandibular defects remains challenging [3]. Autologous bone grafting or microvascular bone transplantation, using donor sites such as the iliac crest, fibula, scapula, or even calvaria, is still the gold standard for reconstruction [2,4]. However, this approach is associated with several drawbacks, including donor site morbidity, extended hospitalization, embolism risks, limited graft availability, intraoperative complications and graft loss [3,4,5]. These limitations have spurred the development of alternative strategies, particularly within the field of bone tissue engineering (BTE) [4,6].

BTE combines biodegradable scaffolds, osteogenic stem cells, and bioactive factors to create three-dimensional constructs that mimic autologous bone [3,4,7,8,9]. These constructs promote bone regeneration through the patient’s self-derived cells, ultimately integrating into the skeletal system [3]. The translation of these promising technologies from the laboratory bench to clinical practice is highly challenging, requiring robust preclinical evidence, especially in animal models, before advancing to clinical application [3].

While immunocompromised rodent models are valuable for studying ectopic bone formation due to their ability to support human tissue engraftment, their small size limits their utility for investigating the orthotopic integration of BTE constructs in load-bearing regions such as the mandible [3]. Large animal models, such as swine, sheep, and dogs, provide a more appropriate platform for evaluating BTE strategies in the context of critical-sized bone defects (CSBDs) [3,10]. These models enable the study of essential factors, such as vascularization within the core of implanted constructs and the biomechanical properties required for functional bone regeneration in load-bearing environments [3,11,12,13].

CSBDs are defined as injuries that surpass the bone’s intrinsic capacity for spontaneous regeneration, requiring surgical intervention for repair [14,15,16]. In mandibular studies, CSBDs are highly variable across species and are influenced by defect size, geometry, and continuity factors. For instance, canine mandibular CSBDs often range from 5 to 25 mm in diameter, with defect volumes between 810 and 2000 mm^3^ [15,17], whereas in pigs, defects exceeding 12–20 mm in diameter and 2800 mm^3^ in volume are considered critical [18]. In primate models, mandibular CSBDs are typically defined as exceeding 20 mm in diameter and 3000 mm^3^ in volume [18]. The periosteum, a critical reservoir of osteoprogenitor cells and growth factors, plays a central role in bone healing. Its removal in segmental defects significantly compromises regenerative outcomes, underscoring the importance of periosteal preservation [19]. Large animal models such as pigs, sheep, and dogs offer anatomical and physiological similarities to humans, providing an essential bridge between preclinical research and clinical applications.

This systematic review aims to assess the current strategies and outcomes of mandibular critical-sized defect reconstruction using TE approaches in large animal models, providing insights into their potential for future clinical applications. Analyzing unique methodologies, including scaffold designs, biomaterials, large animal models, and cell types, provides insights for future clinical applications.

## 2. Materials and Methods

### 2.1. Protocol and Registration

This systematic review was prospectively registered on 27 September 2023 with the International Prospective Register of Systematic Reviews in Health and Social Care (PROSPERO, National Institute for Health Research, York, UK; CRD42023456866). The study adheres to the Preferred Reporting Items for Systematic Reviews and Meta-Analyses (PRISMA) guidelines [20].

### 2.2. Eligibility Criteria

The PICO framework informed the selection criteria for articles [20]. The study population (P) encompassed large animal models (e.g., swine, sheep, dogs), with the intervention (I) focusing on the reconstruction of critical-sized mandibular bone defects through a tissue engineering approach. The comparison (C) involved reconstructions utilizing bone grafts, implantable materials, or untreated groups. The primary outcome (O) was the histological evaluation of newly formed bone tissue, while secondary outcomes included radiographic imaging, biomechanical testing, and qualitative clinical assessments. The inclusion criteria further specified original studies on large animals, mandibular reconstruction using tissue engineering as the primary treatment, critical-sized bone defects exceeding ≥ 20 mm in diameter (no spontaneous healing despite surgical stabilization and requires further surgical intervention [14]), and articles published in English within the last 20 years. For an approach to be classified as tissue-engineered, studies were required to meet at least two of the following criteria: incorporation of a scaffold material, application of osteogenic cells, or use of osteoinductive growth factors. Exclusions comprised in vitro studies, clinical studies, conference abstracts, review papers, letters to the editor, absence of histological analysis, repair through vascularized free flap surgery, studies on periodontal or alveolar regeneration, dental implant studies, and those implementing distraction osteogenesis.

### 2.3. Databases and Search Criteria

Systematic searches were conducted in the MEDLINE/PubMed, Embase, Web of Science, and Cochrane Library databases until 27 September 2024. Search terms were constructed to identify articles related to mandibular reconstruction in large animal models using tissue engineering approaches, while a filter was applied to exclude human studies. The following terms were used to conduct the search: (mandi* OR jaw OR stomatognathic) AND (tissue engineering OR bioengineering OR bioimplant OR biomaterials OR cells OR MSC OR BMSC OR scaffold OR grafts OR growth factor OR bone morphogenic protein OR BMP OR prefabricated OR β-tricalcium phosphate OR β-TCP OR intercellular signaling peptides OR intracellular signaling proteins) AND (large animal model OR sheep OR goat OR porcine OR pig OR swine OR minipig OR dog OR canine OR primates OR chimpanzee OR macaques OR baboon OR cow OR bovine OR horse OR equine) AND (reconstruct* OR segment* OR hemimandibulectomy OR defect OR critical OR defect OR damage).

### 2.4. Data Collection Process

The identified articles were screened independently by two reviewers (E.O. and Y.S.) using the Rayyan.ai research collaboration platform. Conflicting decisions were resolved through a third independent reviewer (F.G.). Preselected articles were downloaded, read in full, and assessed against the inclusion/exclusion criteria by the same reviewers. Disagreements were resolved through discussion with a third reviewer (F.G.).

### 2.5. Data Extraction

Different authors employed a data extraction sheet to systemically extract information from included studies (E.O., A.G., and Y.S.). The data extracted from the articles included the animal model (breed/strain, age, gender, and weight), the characteristics of the study (groups, interventions, tissue engineering approach, defect size, fixation of reconstruction, time points of the studies, complications, and postoperative monitoring), outcome measurements (imaging techniques, histological analysis, histomorphometry, immunohistochemistry, and molecular biology), and the conclusions drawn by the respective authors.

### 2.6. Quality and Risk of Bias Assessment

Compliance with the Animal Research Reporting In Vivo Experiments (ARRIVE) guidelines was assessed to assess the quality of the included studies. The ARRIVE guidelines were developed to guide the reporting of animal studies and thereby improve their reproducibility, inform future researchers, and enable peer review [21]. The ARRIVE guidelines provide a checklist containing 20 items. A grading system adopted by Schwarz et al. [22] was implemented to assess the included studies’ conformity with the ARRIVE guidelines.

Additionally, the risk of bias in the included studies was analyzed according to the Systematic Review Center for Laboratory Animal Experimentation (SYRCLE) tool, which was developed to provide a standardized risk of bias assessment for animal studies [23].

## 3. Results

### 3.1. Search Results

**Figure 1:**
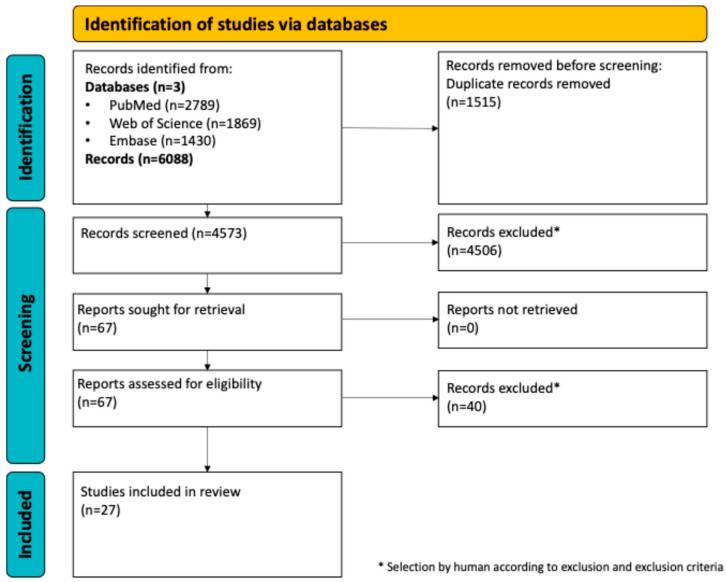
PRISMA flowchart.

The electronic search yielded 6088 articles. This included 2789 through PubMed, 1869 through Web of Science, and 1430 from Embase. In total, 1515 articles were excluded as duplicates and another 4506 were removed after screening the titles and abstracts. The remaining 67 articles underwent full-text review, resulting in 40 exclusions due to inconsistencies with the inclusion/exclusion criteria. Twenty-seven articles were finally included in this systematic review. The PRISMA flow chart demonstrating the screening process can be seen in Figure 1.

### 3.2. Study Design, Animal Model, and Defect Characteristics

The included studies employed a variety of study designs and large animal models to evaluate tissue engineering strategies for mandibular reconstruction, focusing on critical-sized defects.

**Study Design:** Most studies implemented comparative experimental designs involving treatment and control groups. Treatment groups received scaffold-based constructs, often combined with osteogenic cells and/or bioactive molecules, while controls included untreated defects or conventional grafts [24,25]. Follow-up intervals ranged from 4 weeks to 12 months, allowing longitudinal evaluation of bone regeneration [26,27].

**Animal Models:** Porcine models, particularly Göttingen and Yucatan minipigs, were employed in 9 studies [26,28,29,30,31,32,33,34,35] due to their craniofacial similarity to humans [30,32]. Canine models (Beagle, Mongrel) were used in 8 studies [25,36,37,38,39,40,41,42], ovine models in 6, [24,27,43,44,45,46], and non-human primate models (Rhesus Macaque monkeys) in 3 studies [47,48,49]. A single study utilized a caprine model (*n* = 1) [50].

**Defect Characteristics:** Critical-sized defects were primarily located in the body of the mandible, with defect dimensions tailored to species-specific thresholds for spontaneous healing. Defects ranged in size from 20 mm in canine models [25] to 50 mm in porcine models [46]. Segmental defects were the most commonly employed, often created surgically and stabilized using titanium plates or fixation devices to mimic clinical scenarios [28,34]. The absence of the periosteum was a defining feature in many studies, particularly for evaluating the regenerative potential of scaffolds under challenging conditions [24,45].

This section highlights the diversity in animal models and defect designs across the included studies, emphasizing their importance in evaluating the translational potential of tissue-engineered strategies for mandibular reconstruction. Table 1 summarizes detailed information on the animal models, defect characteristics, and study designs.

### 3.3. Bone Tissue Engineering Strategies

The BTE approaches in the included studies typically utilized combinations of biomaterials, cellular components, and bioactive factors to address critical-sized mandibular defects in large animal models.

**Biomaterials:** A diverse range of biomaterials was employed, with β-tricalcium phosphate (β-TCP) being the most commonly used. Pure β-TCP scaffolds were utilized in numerous studies [25,37,42], while others combined β-TCP with additional materials, such as hydroxyapatite or polymers, to enhance their properties [31,32,34]. Poly(lactic-co-glycolic) acid (PLGA) scaffolds, such as those used by Abukawa et al., were also frequently employed [26]. To create porous, biocompatible constructs, PCL scaffolds, including laser-sintered and 3D-printed PCL/β-TCP composites, were explored for their structural and osteoconductive advantages [33,37]. Additionally, naturally occurring materials like coral scaffolds demonstrated promise as osteoconductive and biodegradable matrices [50]. These varied materials underscore the breadth of scaffolding strategies employed in BTE.

**Cellular Components:** Osteogenic cells were integral to many BTE approaches, with BMSCs being the most widely used cell type across studies [24,25,27]. BMSCs were typically harvested and seeded onto scaffolds, enhancing osteogenesis and bone regeneration. In some cases, autologous osteoblasts were employed, such as in studies by Henkel et al. [31], highlighting their potential for direct bone-forming applications. Adipose tissue-derived mesenchymal stem cells (AMSCs) were used in some studies, demonstrating their versatility as an alternative cell source [33,34]. The integration of cellular components across these studies highlights their crucial role in enhancing the osteoinductive potential of biomaterials.

**Growth Factors:** Various studies employed growth factors to enhance osteogenesis and bone healing within tissue-engineered constructs. Recombinant human bone morphogenetic protein-2 (rhBMP-2) was the most commonly used growth factor and was incorporated into scaffolds in several studies [28,29,37,48,49]. These studies demonstrated enhanced bone regeneration and osteoinductive properties, with dose-dependent effects observed in some cases [28]. Another growth factor, recombinant human osteogenic protein-1 (RhOP-1), was used by Wang et al. [40], resulting in improved bone formation and mechanical properties. In vivo bioreactor techniques utilizing local muscle flaps to deliver growth factors were applied in some studies, enhancing vascularization and bone remodeling [43,46,49]. For example, Zhou et al. [49] reported better bone regeneration using the rhBMP-2-incorporated scaffold prefabricated within a latissimus dorsi muscle bioreactor compared to the rhBMP-2-incorporated scaffold alone. Similarly, Tatara et al. [46] demonstrated the efficacy of periosteum-based bioreactors in generating vascularized bone tissue. Platelet-rich plasma (PRP) combined with a polycaprolactone scaffold and porcine adipose-derived stem cells was another effective growth factor strategy, augmenting osteoconductive and osteoinductive properties [33]. Additionally, factor XIII, tested in a single study, did not significantly promote osteogenesis [45]. Overall, growth factors played a pivotal role in enhancing bone regeneration across various tissue-engineered approaches, particularly when combined with advanced scaffolds and cellular components.

The BTE strategies of the included studies can be seen in detail in Table 2.

### 3.4. Outcome Measures

**Histological Analysis:** Histological analysis was the primary outcome parameter to evaluate bone formation, scaffold degradation, and tissue integration across the included studies. Most studies employed hematoxylin and eosin (H&E) staining as a standard method for assessing new bone formation and scaffold biocompatibility [25,26,34]. Additionally, advanced staining techniques, such as Masson’s trichrome [37,41] and Alizarin Red complex staining [40,49], provided detailed insights into mineralization patterns and osteoid deposition. Histomorphometric analyses were conducted in several studies to quantify bone volume and integration rates [28,46]. These assessments consistently highlighted improved bone formation in groups with cellular components or growth factor treatments compared to scaffolds alone.

**Biomechanical Testing:** Biomechanical testing was performed in a subset of studies to evaluate the regenerated bone’s mechanical properties and functional integrity. Three-point bending tests were frequently used to assess the stiffness and load-bearing capacity of tissue-engineered constructs [40,42]. Other methods, such as compressive strength and Young’s modulus testing, provided additional insights into the functional recovery of bone [33,36]. The results from these tests often correlated with histological findings, demonstrating that constructs with enhanced bone regeneration also exhibited superior mechanical properties [29,33].

**Imaging Techniques, Immunohistochemistry, and Molecular Biology:** Imaging modalities, including micro-computed tomography (CT), X-ray radiography, and CT scans, were widely used to evaluate the morphology and volume of regenerated bone [27,34,48]. Micro-CT provided high-resolution assessments of bone structure and scaffold integration, while dual-energy X-ray absorptiometry (DXA) scans were employed in some studies for bone density analysis [25]. Immunohistochemical techniques, such as staining for collagen type I and von Willebrand factor (vWF), were used to assess osteogenesis and vascularization [28,43]. Molecular analyses, including qRT-PCR for osteogenic markers and cytokine profiling, further elucidated the underlying biological processes driving bone regeneration [29,33]. These complementary techniques provided a comprehensive understanding of both the structural and molecular outcomes of mandibular reconstruction.

The methods of outcome assessment are detailed in Table 3.

### 3.5. Assessment of Adherence to the ARRIVE Guidelines

There has been a marked improvement in the reporting of animal experiments, with a growing emphasis on reproducibility and transparency, particularly since the development of the ARRIVE guidelines in 2010. Most papers in the dataset provided detailed and transparent insights into their methodologies, with seven studies offering comprehensive reporting across nearly all guideline categories [27,29,34,38,44,45,46]. These studies demonstrated profound insights into their approach. By contrast, four studies, primarily published before 2010, lacked essential details in their reporting [30,36,41,50].

Several topics outlined in the ARRIVE 2.0 guidelines were consistently addressed across all studies, reflecting an established baseline for good reporting practices. Study design details, including the groups compared (1a), the experimental unit (1b), the sample size (2a), the inclusion and exclusion criteria (3b), the outcome measures (6a), and the experimental procedures (9a, 9b), were thoroughly documented and clearly defined, along with discussions on research objectives (13) and scientific implications (17a).

**Figure 2:**
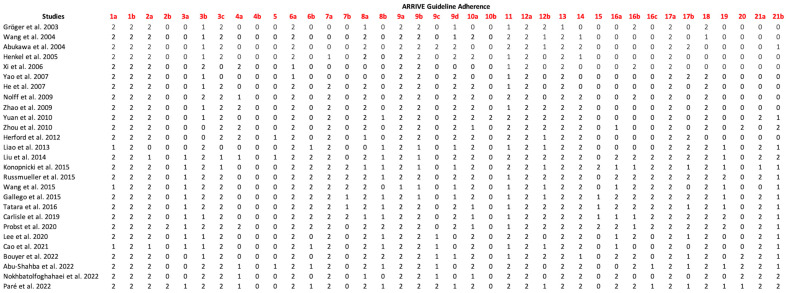
ARRIVE guidelines adherence [9,24,25,26,27,28,29,30,31,32,33,34,35,36,37,39,40,41,42,43,44,45,46,47,48,49,50].

However, some critical aspects were frequently omitted, particularly those related to the rationale behind methodological choices, such as the calculation of the sample size (2b) or the effect size (10b). Techniques to avoid confounding effects (4b) and details about housing and husbandry conditions (15) were also often missing, raising concerns about potential bias and variability in animal welfare. The results of the ARRIVE guidelines evaluation are shown in Figure 2.

### 3.6. Risk of Bias Assessment

**Figure 3:**
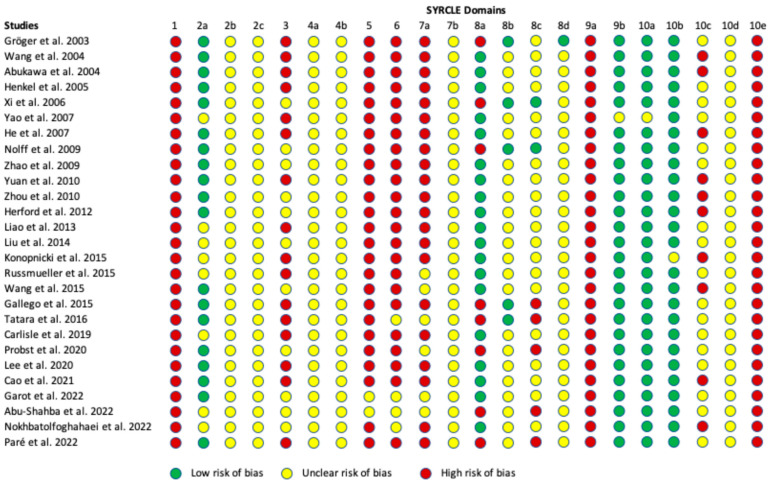
Risk of bias assessment using the SYRCLE tool [9,24,25,26,27,28,29,30,31,32,33,34,35,36,37,39,40,41,42,43,44,45,46,47,48,49,50].

The evaluation of the risk of bias, based on the SYRCLE assessment tool, is shown in Figure 3. Most papers demonstrated a moderate risk level, with four studies providing detailed information to minimize biases in selection, performance, detection, attrition, and reporting [24,25,28,50]. All published reports included the expected outcomes, and there were no indications of “study contamination” or “inappropriate influence of funders.” Additionally, most studies balanced the distribution of relevant baseline characteristics between the intervention and control groups.

However, none of the papers provided detailed study protocols, including information about the allocation/randomization processes or steps for managing dropouts and replacements in the study and control groups. Furthermore, most studies lacked detailed information regarding the blinding of caregivers, investigators, and outcome assessors, raising concerns about potential biases in these areas.

### 3.7. Conclusions Drawn by the Included Studies

As shown in Table 4, tissue engineering approaches have made significant progress in developing constructs for bone regeneration, with a particular focus on mandibular reconstruction. Among the materials studied, β-tricalcium phosphate (β-TCP) emerges as a leading scaffold material due to its ability to support osteogenesis and promote the formation of mature bone structures [24,29,31,32,34,36,37,39,40,45,47,48]. Modifying the scaffold composition further enhances its regenerative capabilities, as demonstrated by HA/β-TCP matrices composed of 60% hydroxyapatite and 40% β-TCP [31,41], which exhibited superior bioactivity and osteoconductive properties compared to conventional hydroxyapatite ceramics. The integration of BMSCs with scaffolds shows remarkable outcomes in bone regeneration. When paired with β-TCP or other advanced scaffolds, BMSCs have promoted uniform radiodensity, active osteoblast and osteocyte activity, and seamless integration with the host bone [24,25,26,27,36,38,42,45,50]. Constructs using GFP-labeled BMSCs have effectively bridged critical-sized defects, achieving biomechanical properties comparable to native bone [42]. Similarly, cryopreserved bone-derived osteoblasts (CBOs) have demonstrated comparable efficacy to fresh osteoblasts, offering a practical solution for bone regeneration, particularly in scenarios involving reduced regenerative capacity due to aging or limited tissue availability [40].

Incorporating rhBMP-2 into scaffolds further enhances osteoinduction, vascularization, and overall bone formation [28,29,37,39,47,48,49]. Additional supplements, such as β-glycerophosphate sodium, dexamethasone, and PRP, have also been shown to enhance osteogenic differentiation and scaffold remodeling, further optimizing regenerative outcomes [33,50]. In vivo muscle-based bioreactors, particularly those incorporating vascularized periosteal flaps, have proven to be an effective strategy for scaffold prefabrication. These bioreactors facilitate vascularization and remodeling while minimizing surgical complexity [39,41,43,46,47,49]. Innovative imaging modalities have been pivotal in evaluating these advancements. Techniques such as 18F-FDG PET/CT [47] and recently developed deep learning-enhanced micro-CT [44] provide reliable and precise methods for tracking bone regeneration, vascularization, and scaffold performance.

## 4. Discussion

This systematic review critically evaluated current BTE strategies for mandibular reconstruction, with an emphasis on approaches targeting CSBDs in large animal models. The review identifies key translational milestones, summarizes major preclinical advancements, and outlines remaining barriers to clinical application. While autologous bone grafting remains the clinical gold standard, it is fraught with challenges, including donor site morbidity, limited graft availability, and long-term complications such as graft resorption [4,51,52,53]. These issues underscore the urgent need for innovative strategies to improve patient outcomes and reduce the associated morbidity. BTE offers a promising alternative, leveraging biodegradable scaffolds, osteogenic cells, and bioactive factors to regenerate functional bone. This systematic review highlights significant advancements in BTE strategies for mandibular reconstruction. These include the development of 3D-printed scaffolds, the incorporation of MSCs, and the use of growth factors. These approaches have significantly improved bone formation, vascularization, and scaffold integration in preclinical models of critical-sized mandibular defects [51,53,54]. However, challenges such as inconsistent vascularization, variability in scaffold degradation, and translational limitations remain hurdles that must be addressed.

### 4.1. Historical Perspective: The Evolution of Mandibular Bone Tissue Engineering

#### 4.1.1. Early Efforts and Initial Challenges

The early phase of BTE focused on using simple biomaterials like β-TCP and HA for mandibular reconstruction. These scaffolds provided basic structural support but suffered from significant limitations, including poor vascularization, incomplete scaffold degradation, and inconsistent bone regeneration [52,53]. The mismatch between scaffold resorption and new bone formation often accumulates residual material, impairing the healing process. Additionally, the lack of biological activity of these materials limited their ability to stimulate robust osteogenesis and integration [52,53,54].

#### 4.1.2. Integration of Cellular Components

Multiple studies have consistently demonstrated that autologous cell seeding—particularly with BMSCs—enhances osteogenic potential, resulting in increased bone volume, improved vascularization, and superior mechanical strength compared to acellular scaffolds.

The inclusion of BMSCs and AMSCs marked a pivotal advancement in BTE. BMSCs enhance osteogenesis by differentiating into osteoblasts and secreting osteogenic factors, while AMSCs provide a versatile and readily available cell source. Multiple studies have consistently demonstrated that autologous cell seeding—particularly with BMSCs—enhances osteogenic potential, resulting in increased bone volume, improved vascularization, and superior mechanical strength compared to acellular scaffolds [38,39,45]. For instance, constructs combining β-TCP scaffolds with BMSCs achieved seamless integration with native bone and superior mechanical stability, paving the way for more effective tissue-engineered constructs [24,34,51,54].

#### 4.1.3. Emergence of Growth Factor Strategies

The addition of bioactive molecules, particularly rhBMP-2, revolutionized the field by enhancing the osteoinductive potential of scaffolds. rhBMP-2 promotes robust and consistent bone regeneration, addressing key limitations of earlier scaffold designs [28,29,37,48]. However, studies have shown that growth factor dose optimization is crucial to minimize complications such as ectopic bone formation and variable efficacy [28]. PRP was another possibility, providing localized delivery of cytokines and growth factors that augmented osteogenic differentiation and scaffold integration; however, its mechanisms remain poorly understood, its composition is highly heterogeneous, and it is more commonly applied in other regenerative contexts, such as the treatment of osteoarthritis [33,52,53,55].

#### 4.1.4. Technological Advancements

Technological innovations such as 3D printing and in vivo bioreactors have transformed scaffold design and prefabrication. Notably, 3D-printed scaffolds allow for precise customization to defect geometry, ensuring an anatomical fit and enhanced functional outcomes [56,57,58,59,60]. Bioreactors that leverage vascularized tissues for scaffold maturation before implantation have significantly improved vascularization and bone regeneration [39,46]. Furthermore, advanced imaging techniques like micro-CT and PET scans provide detailed assessments of scaffold integration, bone volume, and vascularization, refining BTE strategies and improving their reliability [28,34,37,39,44,47,51,53,54].

Histological analysis is vital in validating imaging results and assessing the maturity and quality of newly formed bone in mandibular tissue engineering. Notably, all of the included studies evaluated histological findings, as this was a requirement of the inclusion criteria. By providing detailed insights into bone microstructure and cellular composition, histology complements imaging techniques such as micro-CT, ensuring a more comprehensive evaluation of bone regeneration. This dual approach enhances the reliability of outcome characterization, allowing researchers to better understand the biological processes underlying successful reconstruction. Advanced techniques such as immunohistochemistry and fluoroscopic microscopy provide valuable insights into the rates and maturity of bone formation in mandibular tissue engineering. These methods allow for the detailed analysis of specific markers, such as type I collagen, and serial in vivo radiolabeling to track bone development over time. By offering a deeper understanding of the biological processes involved in regeneration, these techniques complement traditional imaging and histological assessments, providing a more nuanced evaluation of bone quality and the effectiveness of tissue-engineered constructs. Materials used in mandibular reconstruction must withstand the complex biomechanical forces of mastication, including tension, compression, and torsion. Despite this critical requirement, only a limited number of studies have conducted biomechanical testing on newly formed bone [28,34,43,46,47]. The variability in testing approaches and outcome measures across studies has hindered direct comparisons and the development of standardized evaluation methods. A promising approach to assess implant success involves comparing the biomechanical properties of the reconstructed region with those of the contralateral mandible, offering a more reliable benchmark for functional restoration.

There is a critical need for standardized measurements, such as micro-CT and histological analysis, to improve the comparability of outcomes across studies in mandibular tissue engineering [51]. Consistent use of objective metrics, including trabecular thickness, separation, bone mineral density, and percentage of newly formed bone, would enable more reliable comparisons between treatment approaches. Standardizing these methods would enhance the reproducibility of findings and facilitate the development of evidence-based protocols for effective bone regeneration.

### 4.2. Current State of Mandibular Bone Tissue Engineering

#### 4.2.1. Key Achievements

Significant advancements in BTE include integrating cellular and molecular strategies, such as BMSCs and rhBMP-2, with innovative scaffolds. Preclinical studies have demonstrated that these approaches significantly enhance bone formation, vascularization, and biomechanical strength. For example, β-TCP and PCL scaffolds seeded with BMSCs resulted in superior bone integration and mechanical properties compared to controls [28,34,43,47]. Studies employing advanced scaffold designs, such as composite and 3D-printed constructs, have also shown promising outcomes in restoring both structure and function in critical-sized mandibular defects [28,32,37,46,53,54].

#### 4.2.2. Persistent Challenges

Despite these advancements, challenges persist. Scaffold degradation rates often fail to match the pace of new bone formation, leading to residual material accumulation or insufficient structural support during healing. Additionally, variability in the efficacy of growth factors, such as rhBMP-2, has been reported, with dose-dependent inconsistencies and complications such as ectopic bone formation [28]. Hypoxic conditions within larger constructs limit nutrient diffusion and cell viability, posing a critical barrier to effective bone regeneration [51,52,54].

#### 4.2.3. Animal Models

Large animal models play an essential role in mandibular tissue engineering due to their close anatomical, physiological, and biomechanical resemblances to humans. Despite their importance, significant variability in study designs and a lack of standardized approaches often hinder direct comparisons between studies, limiting the ability to draw conclusive insights regarding efficacy [32,61,62]. Regulatory agencies mandate large animal models for preclinical efficacy testing of medical and dental implants in their final human form [32]. Large animal models, including pigs, dogs, sheep, and goats, are preferred for mandibular tissue engineering due to their craniofacial anatomical and biomechanical similarities to humans. These models allow for testing critical-sized defects in a context mimicking human conditions, enabling more clinically relevant outcomes [63,64]. Studies have highlighted that critical-sized mandibular defects pose a significant challenge due to their poor intrinsic healing capacity and the frequent complications associated with internal fixation in large animals, such as plate failures [15,16]. Despite these challenges, large animal models provide a platform to evaluate the regenerative potential of tissue-engineered constructs and their mechanical stability under physiologically relevant forces. Moreover, their size allows for testing human-scale implants and surgical techniques, which is not feasible in small animal models [65,66].

One of the key issues with large animal models is the considerable heterogeneity in study designs, including differences in species/strain selection, defect location, defect size, surgical procedures, and experimental outcome measures. For instance, what constitutes a critical-sized defect varies among species is not consistently defined, which complicates the standardization of preclinical models [15]. Furthermore, while pigs, dogs, and sheep exhibit moderate similarities to human bone properties, differences in bone remodeling, composition, and healing responses remain. For example, Aerssens et al. demonstrated that dog bone is most comparable to human bone in terms of mechanical competence and composition [67]. Small animal models, such as rats and mice, are often used due to their lower costs and widespread availability; however, they fail to replicate the human mandible’s anatomical, biomechanical, and physiological complexities. While useful for initial exploratory studies, these models do not provide the scale or functional relevance required for testing human-sized implants or complex regenerative strategies. By contrast, large animal models allow for testing materials and techniques under conditions that better mimic human clinical scenarios, making them the only way to advance mandibular tissue engineering.

Another limitation is the predominant use of young, healthy, large animals, which does not accurately reflect the demographics of human patients who often present with comorbidities such as advanced age, impaired vascular function, diabetes or radiotherapy in cancer cases. These factors significantly influence bone healing and regenerative outcomes, emphasizing the need for more representative models [68,69,70]. Additionally, immunological differences within and between species, such as varying disease susceptibilities among sheep breeds, further complicate the extrapolation of preclinical findings to humans [71,72]. Selecting an appropriate large animal model is critical for obtaining clinically relevant preclinical data. An ideal model should minimize morbidity, ensure reproducibility, and closely replicate the targeted clinical condition. For mandibular tissue engineering, this includes anatomical and biomechanical similarities and consideration of the biological mechanisms underlying bone regeneration [73]. For example, the reduced efficacy of rhBMP-2 in human orofacial bone regeneration compared to some animal models highlights the importance of selecting models that accurately represent human physiology [74]. Additional considerations include the potential influence of systemic factors, such as age and comorbidities, and the inclusion of strain information for large animals, which is often underreported but has been shown to impact experimental results [72,75]. To improve the utility of large animal models in mandibular tissue engineering, future studies should prioritize standardizing protocols and addressing existing gaps in knowledge. This includes developing age-appropriate and disease-representative models, defining critical-sized defects for specific species, and ensuring consistent strain and breed information reporting. Furthermore, integrating advanced imaging and molecular analysis techniques could provide deeper insights into bone regeneration’s cellular and molecular mechanisms, enhancing the predictive nature of preclinical studies. By addressing these challenges, large animal models can better fulfill their role as a bridge between small animal studies and human clinical trials, ultimately advancing the field of mandibular bone tissue engineering [66,75,76]. The heterogeneity among the included studies—particularly in terms of defect size, animal model, and fixation strategy—presents a notable challenge when attempting to synthesize consistent conclusions. Variability in defect dimensions reflects species-specific anatomical considerations and directly influences the regenerative demands placed on tissue-engineered constructs. For instance, a 20 mm defect in a canine mandible may differ substantially in biomechanical loading and biological response compared to a similarly sized defect in a porcine or ovine model [14,17,18,19]. Likewise, differences in fixation methods, ranging from rigid titanium plates to resorbable systems, alter the mechanical environment of the defect site and can significantly impact outcomes in bone regeneration. The choice of animal model itself introduces further variability, as differences in bone metabolism, immune response, and oral biomechanics can influence both the speed and quality of healing. Despite this heterogeneity, the systematic comparison of methodologies across studies—as provided in this review—offers a valuable framework for identifying the most commonly used and effective parameters. By highlighting trends in defect size, anatomical location, fixation techniques, and outcome measures, this review contributes to the development of more consistent experimental designs. In doing so, it supports the establishment of standardized protocols for large animal models in mandibular tissue engineering, which are essential for improving comparability, reproducibility, and translational potential in future preclinical research.

### 4.3. Future Outlook and Directions

#### 4.3.1. Technological Innovations

Emerging technologies, including biofabrication, smart biomaterials, and personalized scaffolds, hold immense potential to address these challenges. Innovations such as 3D bioprinting enable the creation of complex, patient-specific constructs, while smart biomaterials can dynamically respond to the local microenvironment to promote regeneration [56,57,58,59,60]. Artificial intelligence (AI) offers additional potential for optimizing study design and predicting outcomes [51,54,77]. AI is poised to revolutionize the field of mandibular bone tissue engineering by enhancing every stage of the regenerative workflow—from scaffold design and biomaterial optimization to fabrication, personalized therapy, and clinical translation [51,54,77,78]. In scaffold design, AI-driven algorithms can simulate the interplay between material properties, mechanical loading, and cellular responses, enabling the development of patient-specific constructs with optimized porosity, degradation rates, and mechanical stability. These models can predict scaffold performance under physiological conditions, ensuring a better match with native bone biomechanics and enhancing long-term integration with host tissues [79,80,81,82]. Moreover, AI facilitates the selection and development of next-generation biomaterials by analyzing complex biological datasets to identify favorable chemical interactions and predict biocompatibility outcomes. This capability has accelerated the emergence of smart materials—such as shape-memory polymers and stimuli-responsive composites—that can dynamically adapt to the microenvironment or enable controlled therapeutic release [79,80,82,83]. AI also supports the integration of drug delivery systems into scaffolds, optimizing dosage, timing, and release profiles for enhanced osteoinductive efficacy while minimizing complications [79,80,82]. In fabrication, AI significantly enhances the precision and reproducibility of 3D and 4D printing. By optimizing parameters like printing speed, temperature, and material deposition pattern, AI reduces variability and improves the scalability of scaffold production [79,80,81]. These advancements are especially valuable for manufacturing complex, load-bearing structures like the mandible, where dimensional accuracy and mechanical reliability are critical. The emerging field of 4D printing, enhanced by AI, allows for the fabrication of stimuli-responsive scaffolds that undergo functional transformations post-implantation, offering new opportunities for minimally invasive procedures and patient-specific adaptability [79]. AI’s potential also extends to bridging the translational gap between preclinical research and clinical application. Predictive modeling and advanced simulations can reduce reliance on animal studies by identifying failure points early in the design phase and optimizing scaffold prototypes before clinical testing [81,83]. Furthermore, AI enhances the personalization of regenerative therapies by integrating imaging data, genetic profiles, and clinical history to create individualized treatment strategies. These personalized approaches are particularly relevant for mandibular reconstruction, where anatomical variation and defect complexity often require custom solutions [80,82,83]. Ultimately, AI introduces a paradigm shift toward iterative, data-driven development in bone tissue engineering. Through continuous learning from clinical outcomes and real-world applications, AI can refine scaffold designs and regenerative strategies over time, driving the field toward more effective, reproducible, and patient-centered solutions [81,82]. As computational tools mature and become more integrated into clinical practice, AI will become a cornerstone in the development of next-generation maxillofacial regenerative therapies [78,81,82].

#### 4.3.2. Translational and Clinical Applications

Bridging the gap between preclinical research and clinical application requires large-scale, standardized animal studies and well-designed clinical trials. These efforts should focus on validating scaffold designs, optimizing growth factor delivery, and ensuring consistent outcomes.

Mandibular tissue engineering faces critical limitations that hinder progress and widespread clinical application. One major challenge is the significant heterogeneity in study designs and evaluation methods. Differences in animal models, surgical techniques, materials used, and outcome measurement approaches make it difficult to compare results across studies and draw meaningful conclusions. This lack of standardization limits the reproducibility of findings and delays the development of universally accepted protocols for mandibular reconstruction. Moreover, there is an insufficient understanding of the cellular and molecular mechanisms that underpin successful bone regeneration. The complex interplay between scaffolds, stem cells, and growth factors—known as the regenerative triad—remains poorly characterized. For example, while scaffolds such as calcium phosphates show excellent osteoconductivity, the mechanisms by which they interact with mesenchymal stem cells (MSCs) and bioactive molecules like BMPs to promote bone formation are not fully understood. This knowledge gap limits the ability to optimize scaffold composition, cellular delivery, and growth factor administration to achieve predictable outcomes. Overcoming these limitations requires concerted efforts to standardize experimental protocols and invest in research on the biological processes driving regeneration. A deeper understanding of these mechanisms will enable the identification of the most effective combinations of materials, cells, and signaling molecules, paving the way for reliable and clinically translatable tissue engineering solutions for mandibular reconstruction. From a translational perspective, the evolution of patient-centered innovations across OMS highlights the need to integrate technical advances with patient comfort and outcomes. For example, recent research into conscious sedation protocols for routine procedures such as third molar extractions demonstrates the field’s growing emphasis on individualized care and perioperative experience [84]. Similarly, future clinical applications of BTE must also consider not only regenerative efficacy but also patient safety, operative time, and recovery profiles.

Regulatory and ethical considerations must also be addressed to facilitate the clinical translation of these strategies [51,52]. The lack of standardized protocols for scaffold composition, cell seeding, and growth factor delivery contributes to outcome variability across studies, further hindering clinical translation.

#### 4.3.3. Interdisciplinary Approaches

Advancing BTE will require collaborative efforts across biomaterials science, molecular biology, and surgical research. Interdisciplinary approaches can help to develop comprehensive solutions that address the multifaceted challenges of mandibular reconstruction. Engaging regulatory bodies early in the development process is critical for ensuring the clinical viability of new BTE technologies [51,52].

## 5. Conclusions

Mandibular TE has made significant advancements, with innovations in scaffold design, cellular therapy, and growth factor delivery offering promising solutions to address the limitations of autologous bone grafting. However, persistent challenges, including inconsistent scaffold degradation, vascularization, variability in growth factor efficacy, and a lack of standardized evaluation methods, continue to hinder clinical translation. Due to their anatomical and physiological similarities to humans, large animal models play an essential role in bridging preclinical findings to clinical applications. Yet, variability in study designs and the absence of standardized protocols limit their utility. Future efforts must focus on developing standardized criteria for critical-sized defects, incorporating age-appropriate and disease-representative animal models, and leveraging emerging technologies such as 3D printing and artificial intelligence. By addressing these challenges through interdisciplinary collaboration and regulatory alignment, mandibular TE holds the potential to significantly enhance patient outcomes and transform the field of maxillofacial reconstruction.

## Figures and Tables

**Table 1:** Animal models and defect characteristics [9,24,25,26,27,28,29,30,31,32,33,34,35,36,37,39,40,41,42,43,44,45,46,47,48,49,50].

Studies	Animal Model	Strain/Breed	Age (Years)	Gender	Weight	Size of Defect	Defect Location	Period of Analysis
Gröger et al., 2003	Porcine	Gottingen Minipig	5–6 months	N/A	28–32 kg	20 mm × 10 mm, box shape	Body of mandible	90 and 180 days
Wang et al., 2004	Porcine	Gottingen Minipig	18 months	Female	Ø 43.5 kg	5 cm, box shape	Body of mandible	4, 8, and 12 weeks
Abukawa et al., 2004	Porcine	Yucatan Minipig	6 months	Female	Ø 25 kg	2 cm × 2 cm, box shape	Body and ramus of mandible	6 weeks
Henkel et al., 2005	Porcine	Ellegard Gottingen Minipig	1 year	N/A	Ø 27.4 kg	>5 cm^3^, box shape	Anterior mandible	5 weeks
Xi et al., 2006	Caprine	Guanzhong	1 year	N/A	15–20 kg	25 mm, segmental defect	Body of mandible	4, 8, and 16 weeks
Yao et al., 2007	Canine	N/A	N/A	N/A	N/A	20 mm × 10 mm, box shape	Body of mandible	8 and 12 weeks
He et al., 2007	Canine	Beagle	1–2 years	N/A	10–16 kg	3 cm, segmental defect	Body of mandible	1 and 3 months
Nolff et al., 2009	Ovine	German Blackhead	2–4 years (mean: 3.75)	Female	72.5 ± 7.4 kg	2.7 cm × 1.5 cm, triangular	Body of mandible	12 weeks
Zhao et al., 2009	Canine	Mongrel	18 months	Male	15–20 kg	20 mm × 10 mm, box shape	Body of mandible	1, 3, 6, and 12 months
Yuan et al., 2010	Canine	Mongrel	16 months	N/A	19.7 kg	3 cm, segmental	Body of mandible	4, 12, 26, and 32 weeks
Zhou et al., 2010	Primate	Rhesus	6–9 years	Male	6–12 kg	20 mm × 10 mm × 15 mm (3 cm^3^)	Body of mandible	26 weeks
Herford et al., 2012	Primate	Rhesus Macaque	“Skeletally mature”	Male	N/A	2.5 cm	Body of mandible	6 months
Liao et al., 2013	Porcine	N/A	N/A	N/A	N/A	3 cm × 3 cm	Body of mandible	6 months
Liu et al., 2014	Canine	Beagle	2 years	Female	10 ± 3 kg	Full condylectomy	Body of mandible, ramus, and condyle	4, 12, 24, and 48 weeks
Konopnicki et al., 2015	Porcine	Yucatan Minipig	N/A	N/A	N/A	20 mm × 20 mm	Body of mandible	8 weeks
Russmueller et al., 2015	Ovine	N/A	Skeletally nature	Female	60–80 kg	30 mm × 30 mm	Angle of mandible	12 weeks
Wang et al., 2015	Canine	Beagle	12–18 months	Female	Average 12.5 kg	30 mm, segmental defect	Body of mandible	12 months
Gallego et al., 2015	Ovine	Laxta Asturian	12–15 months	Female	57.3–64.8 kg	30 mm, segmental defect	Parasymphysis	12 and 32 weeks
Tatara et al., 2019	Ovine	Dorper	4–6 months	Female	40.4 ± 7.3 kg	2 cm, box shaped	Body of mandible	9 and 21 weeks
Carlisle et al., 2019	Porcine	Sinclair Minipig	>12 months	Female	N/A	2 cm, segmental defect	Body of mandible	4, 8, and 12 weeks
Probst et al., 2020	Porcine	Munchener Trollschweine	12–14 months	Mixed gender	Mean: 85 kg	3 cm × 1 cm × 2 cm, box shape	Body and angle of mandible	12 weeks
Lee et al., 2020	Canine	Beagle	12–15 months	Male	12.5 kg	20 mm × 10 mm × 10 mm, box shaped	Body of mandible	12 weeks
Cao et al., 2021	Primate	Rhesus	6–9 years	Male	6–12 kg	20 mm × 15 mm × 10 mm, segmental defect	Body of mandible	12 weeks
Bouyer et al., 2022	Porcine	Minipigs	>24 months	Female	43–69.5 kg	4 cm × 3 cm, Box shaped	Body and angle of mandible	16, 30, 51, and 91 days
Abu-Shahba et al., 2022	Ovine	Texel and Crossbred	24–35 months	Female	51–65 kg	29 (±2) mm × 18 (±1) mm	Angle of mandible	13 and 23 weeks
Nokhbatolfoghahaei et al., 2022	Canine	Mongrel	N/A	N/A	15–25 kg	25 mm × 10 mm × 8 mm	Posterior mandible	12 weeks
Paré et al., 2022	Ovine	Vendean	4–7 years	Female	75–84 kg	35 mm × 55 mm, segmental defect	Angle of mandible	3, 5, and 12 months

N/A, not applicable/not performed/not described.

**Table 2:** Bone tissue engineering strategies [9,24,25,26,27,28,29,30,31,32,33,34,35,36,37,39,40,41,42,43,44,45,46,47,48,49,50].

	Tissue Engineering Approach			
Studies	Groups	Biomaterial	Cells	Growth Factors/Bioreactors
Gröger et al., 2003	Treated vs. Non-treated Groups 1 (n = 4) and 2 (n = 2)	3D polymer Fibrin-fleece scaffold (Ethisorb 510^®,^, Ethicon, Raritan, NJ, USA)	Unspecified bone tissue derived cells, treated with osteogenic media	N/A
Wang et al., 2004	Group 1: Placebo-treated (n = 1). Group 2: RhOP-1 treated (n = 4)	Type I collagen bone matrix (ground)	N/A	Recombinant human osteogenic protein-1 (RhOP-1)
Abukawa et al., 2004	Constructs (n = 2), Control (n = 1), Empty (n = 1)	Poly-lactic-co-glycolic (PGLA) scaffold	Autologous BMSCs	Osteogenic differentiation media + ROBS
Henkel et al., 2005	Groups with control/osteoblasts/scaffold alone/scaffold + osteoblasts (n = 4 each)	60% Hydroxyapatite, 40% β-TCP matrix	Autologous osteoblasts	N/A
Xi et al., 2006	Experimental vs. Control (scaffold alone) (n = 10 in total)	Coral scaffold	Autologous BMSCs	N/A
Yao et al., 2007	Animals received in vivo tissue-engineered bone/no control group (n = 3)	Ca-P ceramic	N/A	In vivo bioreactor (femoral muscle)
He et al., 2007	β-TCP scaffold + BMSCs (n = 3) β-TCP scaffold alone (n = 3)	β-TCP	3rd generation BMSCs	Osteogenic differentiation medium
Nolff et al., 2009	Group 1: β-TCP composite (n = 6); Group 2: β-TCP composite with bone marrow and cancellous bone (n = 6)	β-TCP cylinders	Bone marrow stromal cells (BMSCs) + mozelized cancellous bone	N/A
Zhao et al., 2009	5 groups (n = 4 each), SS/mSS with/without BMSCs	Silk fibroin scaffold; apatite-coated silk fibroin scaffold;	BMSCs (2–3 passage)	N/A
Yuan et al., 2010	1. Group: BMSCs + coral cuboids (n = 12) 2. Group: coral cuboid alone (n = 12)	Coral cuboids (natural coral)	BMSCs	Osteogenic differentiation medium
Zhou et al., 2010	DFDBA-BMP, CHA-BMP, DFDBA, CHA (n = 3 per group)	Demineralized freeze-dried bone allograft or coralline hydroxyapatite	N/A (latissimus dorsi auto-bioreactor)	rhBMP-2
Herford et al., 2012	5 groups with varying rhBMP-2/ACS + CRM (n = varies/26 defects total)	Absorbable collagen sponge (ACS), compression-resistant matrix (CRM) (HA + β-TCP)	N/A	rhBMP-2
Liao et al., 2013	Group A: PCL alone Group B: PCL/PRP/PASCs	Laser-sintered porous polycaprolactone (PCL) scaffold	PASCs	PRP
Liu et al., 2014	Control/Experimental groups (n = 30 total)	Allogenic freeze-dried scaffold	BMSCs	N/A
Konopnicki et al., 2015	Seeded Scaffold (n = 3) Unseeded scaffold (n = 3) Empty defects (n = 3)	50% β-TCP/50% PCL scaffold	Autologous bone marrow-derived osteoblasts	N/A
Russmueller et al., 2015	ChronOS/bone marrow (n = 6) ChronOS/factor XIII (n = 6) ChonOS/venous blood (n = 6)	Polylactide scaffold containing a tricalcium phosphate biomaterial (chronOS^®^, DePuy Synthes, Warsaw, IN, USA)	BMSCs	Factor XIII
Wang et al., 2015	CBOs/β-TCP (n = 4) FBOs/β-TCP (n = 4) β-TCP (n = 4) Autogenous mandibular segment (n = 4)	β-TCP scaffold	Cryo-preserved osteoblasts (CBOs)and fresh bone-derived osteoblasts (FBOs)	N/A
Gallego et al., 2015	Scaffold alone (n = 5) Scaffold/BMSCs (n = 8)	Serum-based, custom mold	BMSCs	N/A
Tatara et al., 2019	Autograft (AG) (n = 15) Synthetic graft (SG) (n = 9)	PMMA, carboxymethylcellulose-gel	N/A	In vivo bioreactor (rib periosteum)
Carlisle et al., 2019	Polyurethane (PUR) +rhBMP-2 (n = 6); Untreated (n = 6)	Polyurethane (PUR) + HA/β-tricalcium phosphate scaffold	N/A	rhBMP-2
Probst et al., 2020	Scaffold + pADCs (n = 8) Empty scaffold (n = 8)	Tri-calcium phosphate infiltrated with polymer (TCP-PLGA)	Pig adipose-derived stem cells (pADSCs)	N/A
Lee et al., 2020	No treatment (n = 4) PCL/β-TCP (n = 4) PCL/β-TCP/rhBMP-2 (n = 4) PCL/β-TCP/ABPs (n = 4)	PCL/β-TCP	Autologous bone particles (ABPs)	rhBMP-2
Cao et al., 2021	5 groups (n = 3 each), TCP/PLGA variations	TCP, PLGA/TCP scaffolds	N/A	rhBMP-2; autologous bioreactor (Latissimus dorsi)
Bouyer et al., 2022	Autologous bone graft (n = 4) PLA scaffold BMP-2 coating (varying dose) (n = varies)	PLA scaffold, 24 alternative polyelectrolyte films (HA and PLL)	N/A	rhBMP-2
Abu-Shahba et al., 2022	Control, M, MP, MVP (n = 5 per group)	Bovine-derived mineral matrix, reinforced with resorbable poly(lactic-co-caprolactone) copolymer and RGD-exposing collagen fragments for surface activation	N/A	Autologous bioreactor (M, MP, MVP)
Nokhbatolfoghahaei et al., 2022	βTCP, βTCP/rhBMP2, βTCP/MSCs, PCL/βTCP, PCL/βTCP/rhBMP2, and PCL/βTCP/MSCs (n = 4 each)	FDM 3D-printed PCL/βTCP; foam-cast βTCP	Adipose-derived MSCs	rhBMP-2; autologous bioreactor (Masseter)
Paré et al., 2022	BCP + TBM (n = 6) VBT control (n = 6)	BCP bioceramic implants	Total bone marrow (TBM)	Perfused by an arteriovenous loop (in loco bioreactor)

N/A, not applicable/not performed; RhOP-1, recombinant human osteogenic protein-1; ROBS, rotational oxygen-permeable bioreactor system; Ca-P, calcium and phosphate; β-TCP, beta-tricalcium phosphate; BMSCs, bone marrow-derived mesenchymal stem cells; SS, silk fibroin scaffold; mSS, apatite-coated silk fibroin scaffold; DFDBA, demineralized freeze-dried bone allograft; CHA, coralline hydroxyapatite; DFDBA-BMP, rhBMP-2-incorporated DFDBA; CHA-BMP, rhBMP-2-incorporated CHA; PASCs, porcine adipose-derived stem cells; PRP, platelet-rich plasma; PCL, laser-sintered porous polycaprolactone; PMMA, polymethylmethacrylate; PCL, poly caprolactone; ABPs, autogenous bone particles; PLA, poly(L-lactide); PLL, poly(L-lysine); M, intramuscular pouch in the rostral part of brachiocephalic muscle; MP, pericranial nonvascularized graft with the muscular pouch; MVP, pericranial vascularized flap with the muscular pouch, FDM, fused deposition modeling; MSCs, mesenchymal stem cells; BCP, biphasic calcium phosphate; VBT, vascularized bone transplant.

**Table 3:** Outcome assessment [9,24,25,26,27,28,29,30,31,32,33,34,35,36,37,39,40,41,42,43,44,45,46,47,48,49,50].

	Outcome Parameters			
Studies	Imaging	Biomechanical Testing	Histology/Histomorphometry	Immunohistochemistry/Molecular Biology
Gröger et al., 2003	X-ray radiography	N/A	Masson’s trichrome staining	N/A
Wang et al., 2004	X-ray radiography, CT scan	Three-point bending test	Toluidine blue staining	N/A
Abukawa et al., 2004	X-ray radiography	N/A	Hematoxylin and eosin staining	N/A
Henkel et al., 2005	X-ray radiography	N/A	Hematoxylin and eosin staining	N/A
Xi et al., 2006	X-ray radiography, scanning electron microscope	N/A	Hematoxylin and eosin staining	N/A
Yao et al., 2007	X-ray radiography	N/A	Hematoxylin and eosin staining, Masson’s trichrome staining	Tetracycline fluorescence labeling for histomorphometry analysis
He et al., 2007	X-ray radiography, CT scan	Compression, stress, and energy tests	Hematoxylin and Masson’s staining	N/A
Nolff et al., 2009	N/A	N/A	Alizarin and methylene blue staining, histomorphometry analysis performed on histology sections	N/A
Zhao et al., 2009	X-ray radiography, CT scan, DXA scan	N/A	Hematoxylin and eosin staining, histomorphometry analysis performed on histology sections	N/A
Yuan et al., 2010	X-ray radiography, micro-CT scan	Three-point bending test	Van Gieson’s picrofuchsine stain	N/A
Zhou et al., 2010	X-ray radiography, angiography	N/A	Hematoxylin and eosin staining	Alizarin complexion, tetracycline, xylenol orange, and calcein for histomorphometric analysis
Herford et al., 2012	X-ray radiography, micro CT	N/A	Hematoxylin and eosin staining, Masson’s trichrome staining	Histomorphometry analysis performed on histology sections, toluidine blue to evaluate new bone formation
Liao et al., 2013	3D-CT	Compressive Young’s modulus test	Masson’s trichrome staining	Immunohistochemistry with collagen type I and osteocalcin, qRT-PCR assessment for alkaline phosphatase activity
Liu et al., 2014	3D-CT, micro-CT, 36-XR dual energy X-ray absorptiometry scan	N/A	Hematoxylin and eosin staining	Histomorphometry analysis performed on histology sections
Konopnicki et al., 2015	CD 31 immunofluorescence	N/A	Hematoxylin and eosin staining, nuclear staining with 4′,6-diamidino-2-phenylindole stain	Histomorphometry analysis performed on histology sections, primary anti-pig CD31 anti- body immunohistochemistry stain (for angiogenesis)
Russmueller et al., 2015	X-ray radiography	N/A	1% thionine stain	Histomorphometry analysis performed on histology sections
Wang et al., 2015	X-ray radiography, CT	N/A	Fluorescent labeling under confocal laser scanning microscope, tetracycline hydrochloride, calcein, alizarin, and calcein blue	Tetracycline hydrochloride, calcein, alizarin, and calcein blue for histomorphometry, diaminobenzidine substrate counterstained with hematoxylin for immunohistochem
Gallego 2015	CT, micro-CT	N/A	Hematoxylin and eosin staining, Masson’s trichrome staining	N/A
Tatara et al., 2019	Micro-CT,	Compression load test	Methylene blue/basic fuchsin stain	Histomorphometry analysis performed on histology sections
Carlisle et al., 2019	Micro-CT	N/A	van Gieson’s picrofuchsin stain	Osteogenesis and rhBMP-2 release cytokine profile analysis
Probst et al., 2020	Micro-CT	N/A	Hematoxylin and eosin staining	Osteocalcin immunostaining
Lee et al., 2020	Micro-CT	N/A	Hematoxylin and eosin staining, Masson’s trichrome staining	Histomorphometry analysis performed on histology sections
Cao et al., 2021	PET and CT imaging, angiography	Uniaxial compressive testing	Hematoxylin and eosin staining	Histomorphometry analysis performed on histology sections
Bouyer et al., 2022	CT scan, micro-CT analysis	N/A	Sanderson’s rapid stain and Van Gieson’s stain	Histomorphometry analysis performed on histology sections
Abu-Shahba et al., 2022	CT, CT angiography, micro-CT	N/A	Hematoxylin and eosin staining, Masson’s trichrome staining, picrosirius red, reticulin, and Movat’s pentachrome staining	Immunohistochemical (IHC) staining using anti-von Willebrand factor (vWF) antibody to assess vascularization, histomorphometry analysis performed on histology sections
Nokhbatolfoghahaei et al., 2022	CT scan	N/A	Hematoxylin and eosin staining	Histomorphometry analysis performed on histology sections
Paré et al., 2022	CT scan, micro-CT, scanning electron microscope	N/A	Hematoxylin and eosin stain, Safranin stain, Movat’s pentachrome stain	N/A

N/A, not applicable/not performed; DXA, bone density scan.

**Table 4:** Conclusions Drawn by the Included Studies [9,24,25,26,27,28,29,30,31,32,33,34,35,36,37,39,40,41,42,43,44,45,46,47,48,49,50].

Studies	Take-Home-Massages
Gröger et al., 2003	**Enhanced Radiodensity and Tissue Integration**: Increased radiodensity and calcification were observed in defects filled with cell-fibrin-fleece constructs compared to untreated controls after 90 and 180 days, with complete integration at defect borders and seamless host–implant transitions. **Promising Approach for Mandibular Augmentation**: The combination of periosteal cells and polymer fleece facilitated membranous bone formation without acute inflammation, suggesting clinical potential for mandibular augmentation. **Phenotype Shift and Improved Healing**: Light microscopy showed a shift to cuboid osteoblast-like cells, along with vascularization and calcification over time, attributed to the short degradation time and optimized fleece structures.
Wang et al., 2004	**Effective Bone Regeneration with CMC-Stabilized Collagen Matrix**: The study demonstrated successful regeneration of a 5 cm mandibular defect in Göttingen miniature pigs using rhOP-1 delivered with a CMC-stabilized collagen type I matrix, filling the defect with sufficient bone volume without foreign body reaction. **Enhanced Bone Formation and Space-Keeping Properties**: The rhOP-1-treated group showed increased bone volume, density, and mineralization, with complete defect filling and good plasticity, although mechanical stress resistance was 25% lower than the control side. **Species-Specific Optimization and Potential Limitations**: The optimal rhOP-1 concentration varies by species and defect, with cartilage and fibrous tissue observed under reconstruction plates potentially due to impaired rhOP-1 interactions with stem cells in surrounding tissues.
Abukawa et al., 2004	**Successful Reconstruction with Tissue-Engineered Constructs**: Porcine mandibular defects were effectively reconstructed using autologous mesenchymal stem cells (MSCs) cultured on a biodegradable polymer scaffold, resulting in hard, noncompressible tissue that was indistinguishable from native bone, with complete defect bridging. **Bone Formation and Vascularization**: The tissue-engineered constructs promoted uniform radiodensity, osteoblast and osteocyte presence, bone trabeculae, and primitive blood vessels throughout the defects, contrasting with fibrous tissue observed in controls. **Integration and Structural Similarity**: Bone reconstructed with tissue-engineered constructs showed seamless integration with adjacent native bone, with indistinct margins and character comparable to natural bone, demonstrating the potential of scaffold designs with varied pore sizes for effective jaw reconstruction.
Henkel et al., 2005	**Superior Bone Formation with Biomatrix Alone**: The biomatrix group without osteoblasts showed the highest new bone formation rate, filling 73% of the defect, with biodegradation matching the pace of new bone deposition, outperforming groups with osteoblast transplantation. **Effective Osteoconductive Properties**: The HA-bTCP matrix (60% hydroxyapatite, 40% beta-tricalcium phosphate) demonstrated high bioactivity and osteoconductive capabilities, surpassing conventional hydroxyapatite ceramics as a temporary bone replacement material. **Limited Impact of Osteoblast Transplantation**: Adding autologous osteoblasts to the biomatrix did not enhance bone production compared to controls.
Xi et al., 2006	**Successful Bone Regeneration with Coral and Titanium Reinforcement**: Histological analysis showed new bone formation on the surface and within the pores of natural coral, with grafts fully restored after 16 weeks. Titanium reticulum reinforcement enhanced mandibular defect restoration with high biocompatibility and minimal stress shielding. **Coral Microstructure and Bone Healing**: Natural coral’s trabecular-like microstructure supported osteogenesis, with smooth, red bone tissue covering graft surfaces in the cell seeding group, although bone healing was limited to areas outside the titanium reticulum due to periosteal proliferation. **Enhanced Osteogenic Phenotype with Supplements**: Adding bone morphogenetic protein, β-glycerophosphate sodium, and dexamethasone to the medium improved the osteogenic properties of the cells, aiding in reconstructing segmental mandibular defects with tissue-engineered bone.
Yao et al., 2007	**Successful Integration and Bone Regeneration**: The in vivo tissue-engineered (TE) bone graft integrated with host bone, supported active bone regeneration, and restored mandibular shape without infection or inflammation, demonstrating feasibility for box-like mandibular defect reconstruction. **Enhanced Bone Properties with Ca-P Ceramics**: Calcium phosphate ceramics (60% HA/40% α-TCP, 60% porosity) facilitated osteoconductivity and osteoinduction, with new bone forming into ceramic pores, maturing with trabeculae and Haversian systems, and modifying ceramic’s poor biomechanical properties. **Bioactivity and Host Participation**: The TE bone graft, carrying living cells and a good blood supply, participated in host bone metabolism, achieving biomechanical properties and bioactivity comparable to autografts.
He et al., 2007	**Enhanced Bone Regeneration and Biomechanical Strength**: Tissue-engineered constructs combining bone marrow stromal cells and a 3D β-tricalcium phosphate scaffold significantly improved bone formation, radiodensity, and biomechanical properties, minimizing donor site morbidity while offering a customizable solution for complex 3D mandibular defects. **Histological and Structural Advantages**: New bone formation, osteoblast activity, and cartilage were observed in the scaffold’s central sections after 3 months, with the engineered graft precisely shaped to mimic the lost bone using computer-aided design.
Nolff et al., 2009	**Effective Bone Healing with B-TCP Composite**: The beta-TCP composite loaded with bone marrow and cancellous bone (B-TCPB/BM/CB) effectively healed critical-sized mandibular defects, showing significantly higher bone formation and osteointegration compared to B-TCP alone, with dense lamellar bone bridging the defects. **Clinical Potential and Surgical Advantages**: The B-TCP composite offers a promising alternative to autografts for mandibular reconstruction, enabling table-side preparation with the patient’s own cells, avoiding cell culture or expansion, and showing potential for various clinical applications.
Zhao et al., 2009	**Effective Repair of Mandibular Defects**: The combination of bMSCs with an apatite-coated silk scaffold (bMSCs/mSS) completely repaired canine mandibular border defects within 12 months, achieving bone mineral densities comparable to normal mandibles and reconstructing the mandibular contour seamlessly with native bone. **Enhanced Osteoconductive Environment**: The premineralized silk fibroin scaffold provided an osteoconductive matrix for bMSCs, promoting differentiation into osteoblasts, extracellular matrix secretion, and preventing fibrillar tissue infiltration, resulting in substantial new bone formation and vascularization.
Yuan et al., 2010	**Effective Repair and Bone Integration**: GFP-labeled BMSCs on beta-TCP coral scaffolds successfully repaired 30 mm critical-sized mandibular defects in 32 weeks, achieving bony union, smooth remodeling, and biomechanical properties comparable to normal mandibles, ensuring long-term stability and function. **Scaffold Degradation and Osteogenesis**: Coral scaffolds demonstrated an ideal degradation rate, with reduced residual volumes over time, facilitating new bone formation as BMSCs differentiated into osteoblast-like cells and integrated with endogenous MSCs.
Zhou et al., 2010	**Prefabricated Bone Flaps with rhBMP-2 Show Superior Regeneration**: Combining prefabricated tissue-engineered bone flaps with an rhBMP-2-incorporated CHA scaffold effectively reconstructs mandibular critical-sized defects, achieving robust bone regeneration and structural integrity. **Key Role of rhBMP-2 in Osteoinduction**: The integration of rhBMP-2 enhances osteoinductive properties, promoting homogeneous bone formation, vascularization, and functional remodeling in mandibular reconstructions. **Long-Term Structural and Functional Success**: Mandibular defects reconstructed with rhBMP-2-incorporated CHA scaffolds demonstrated bone morphology, density, and mechanical properties comparable to native mandibles, emphasizing the potential for clinical applications.
Herford et al., 2012	**Superior Bone Formation with CRM and rhBMP-2**: The combination of CRM and rhBMP-2 achieved significantly greater bone density and reduced voids, leading to effective mandibular defect repair compared to ACS-based carriers. **Effective Space Maintenance**: The compression-resistant properties of CRM preserved the defect structure, supporting consistent and robust bone regeneration. **Critical Role of rhBMP-2 and Carrier Synergy**: The optimized release and high-dose delivery of rhBMP-2 facilitated early osteoinduction, highlighting the importance of carrier properties in enhancing bone regeneration outcomes.
Liao et al., 2013	**Enhanced Bone Formation**: The PCL/PRP/PASCs construct supported robust new bone formation with increased density and compact structure, crucial for successful mandibular defect repair. **Synergistic Osteoinduction**: The combination of PRP and PASCs within the PCL scaffold promoted osteogenic differentiation, contributing to effective bone regeneration. **Effective Scaffold Integration**: The construct’s interconnected porous structure facilitated seamless integration with surrounding bone tissue, promoting uniform bone growth throughout the scaffold.
Liu et al., 2014	**Enhanced and Accelerated Bone Remodeling**: Autologous MSCs accelerated the absorption of allogenic scaffolds and facilitated their complete replacement with new bone within 48 weeks, promoting trabecular bone formation, Haversian canal expansion, and significantly improving bone mineral density and micro-architecture. **Need for Optimization**: The prevalence of fibrous ossification and postoperative infections highlights the need for additional growth factors and strategies to improve bone quality and reduce complications.
Konopnicki et al., 2015	**Effective Bone Formation**: 3D-printed b-TCP and PCL scaffolds seeded with pBMPCs demonstrated robust bone penetration, with significantly higher bone formation in the center of constructs compared to unseeded scaffolds. **Angiogenesis and Scaffold Resorption**: Enhanced CD31 expression and vascularization were observed in the constructs, particularly in areas of new bone, facilitating scaffold resorption and bone integration. **Critical Role of Early Implantation**: Early implantation supports efficient cell penetration, collagen deposition, and extracellular matrix formation, optimizing the healing process and promoting bony architecture development.
Russmueller et al., 2015	**Superior Performance of Autologous Bone Marrow**: Polylactide scaffolds combined with tricalcium phosphate biomaterial and autologous bone marrow demonstrated robust bone regeneration across all regions of interest, preserving scaffold structure and achieving consistent osteoconductive bone formation. **Limited Efficacy of Factor XIII**: Contrary to prior studies, coagulation factor XIII failed to enhance bone regeneration, showing performance comparable to blood-based controls and significant scaffold deformation.
Wang et al., 2015	**Effective Bone Regeneration with CBOs and β-TCP**: Tissue-engineered bone using cryopreserved bone-derived osteoblasts (CBOs) combined with β-TCP successfully repaired critical-sized segmental mandibular defects, promoting bone mineralization and deposition comparable to fresh bone-derived osteoblasts (FBOs). **Clinical Potential of CBOs**: The use of CBOs offers a practical solution for large-volume bone regeneration, addressing limitations of tissue banking and enabling reconstruction even in cases of reduced regenerative capacity due to aging. **Immunohistochemical Validation**: Intensive osteocalcin expression in the bone matrix of CBO and FBO groups confirmed active bone formation and integration, further supporting the osteogenic potential of these constructs.
Gallego et al., 2015	**Improved Bone Quality with BM-MSCs:** Segmental mandibular defects repaired using the serum scaffold seeded with autologous BM-MSCs exhibited significantly enhanced bone quality, with BMD, BV/TV, trabecular thickness (TbTh), and trabecular number (TbN) all significantly higher than in the control group at 32 weeks. The newly formed bone in the experimental group was similar to native bone **Localized Bone Formation**: Ossification was most advanced in the central area of the defect in the BM-MSCs-seeded scaffold group, demonstrating a high degree of mineralization and osteon formation **Faster and More Consistent Bone Union**: The BM-MSCs-seeded scaffold group achieved earlier and more consistent bony union compared to the control group.
Tatara et al., 2019	**Successful Use of 3D-Printed Bioreactors for Mandibular Reconstruction**: A total of 83% of 3D-printed in vivo bioreactors generated mineralized tissue suitable for reconstructing large mandibular defects in sheep, demonstrating their potential for creating autologous vascularized bone free tissue flaps. **Superior Bone Quality with AG Scaffolds**: AG scaffolds supported the formation of more mature bone tissue with mechanical properties closer to native bone, outperforming SG scaffolds in generating bone suitable for reconstructive purposes. **Effective Space Maintenance**: Space maintainers integrated well with local soft tissue and successfully preserved the defect area, allowing for the generation of customized bone grafts that matched the geometry of the mandibular defect.
Carlisle et al., 2019	**Enhanced Bone Regeneration with Low-Dose rhBMP-2**: Treatment with low-dose rhBMP-2 delivered through a PUR composite with calcium phosphate granules significantly enhanced bone regeneration, leading to complete bone bridging in mandibular continuity defects within 12 weeks, as evidenced by increased bone volume and mineral density. **Localized Healing Response**: The regenerative treatment elicited a localized inflammatory response with increased levels of cytokines such as IL-1ra and IL-6 at early time points, without systemic inflammation or excessive cytokine levels, indicating a controlled and safe therapeutic effect. **Localized Delivery and Safety**: The PUR scaffold enabled localized delivery of rhBMP-2 without detectable systemic absorption, reducing the risk of systemic side effects and ensuring a controlled release for effective bone regeneration.
Probst et al., 2020	**Enhanced Bone Regeneration with ADSCs**: ADSCs-seeded TCP-PLGA scaffolds demonstrated significantly improved bone volume and osteocalcin deposition compared to non-seeded scaffolds after 12 weeks, indicating the osteogenic potential of ADSCs in large mandibular defect repair. **Challenges with Hypoxia in Scaffold Centers**: Despite the interconnected macroporous design of the scaffold facilitating vascular ingrowth, hypoxic conditions in the scaffold center hindered complete bone regeneration. **Scaffold Integration and Stability**: The TCP-PLGA scaffold was well integrated into the defect area and fixable with titanium screws, although brittleness and the need for improved mechanical properties remain limitations.
Lee et al., 2020	**Enhanced Bone Regeneration with Additives**: PCL/β-TCP scaffolds loaded with rhBMP-2 or autogenous bone particles (ABP) significantly improved bone regeneration compared to the control or PCL/β-TCP scaffolds alone. Micro-CT analysis revealed that the rhBMP-2-loaded scaffold group generated the highest bone volume among the groups, followed by the ABP-loaded scaffold group. **Limitations in Clinical Application**: Despite improved bone formation in experimental groups, the volume of newly formed bone was insufficient for clinical application. Periosteal resection and the lower dose of rhBMP-2 contributed to these suboptimal outcomes, highlighting the need for further optimization of growth factor dosage and scaffold design **Scaffold Design and Stability**: The 3D-printed PCL/β-TCP scaffold, with its heterogeneous pore sizes and additional wing structures, provided stable screw fixation and allowed for proper integration within mandibular defects
Cao et al., 2021	**Superior Stability and Osteoconductivity of TCP Scaffolds**: β-TCP scaffolds demonstrated significantly superior in vivo stability, mechanical strength, and osteoconductivity compared to PLGA/TCP scaffolds, retaining their 3D architecture and porous structure even after prolonged implantation. **Enhanced Bone Regeneration with rhBMP-2 Coating**: TCP scaffolds coated with rhBMP-2 showed a notable increase in bone volume and mineralization at both ectopic and orthotopic implantation sites. Prefabricated rhBMP-2-coated TCP scaffolds (P-TCP-BMP) achieved significantly better outcomes in bone regeneration and structural integration than directly implanted rhBMP-2-coated TCP scaffolds (S-TCP-BMP). **Utility of 18F-FDG PET/CT in Monitoring Regeneration**: 18F-FDG PET/CT provided a reliable method for tracking bone regeneration and vascularization, highlighting higher uptake in the rhBMP-2-coated TCP scaffold, correlating with enhanced scaffold performance.
Bouyer et al., 2022	**BMP-2 Dose-Dependent Bone Regeneration and Maturity:** BMP-2 doses significantly influenced the rate, quality, and maturity of bone regeneration. Higher BMP-2 doses (e.g., BMP110) led to significantly greater bone volume and mineralization, producing mature bone with Haversian canals and robust integration between host and regenerated bone. These natural bone-like connections enhanced mechanical stability, mimicking the structure of native bone. Repair kinetics were also dose dependent, with slower repair observed for higher BMP-2 doses. **Enhanced Performance of EDC30 Films:** EDC30 films provided superior BMP-2 adsorption and sustained release compared to EDC70 films, leading to more robust and predictable bone regeneration. **Comparable Outcomes and Safety with BMP-2-Loaded 3D-Printed Scaffolds**: BMP-2-loaded 3D-printed PLA scaffolds demonstrated bone regeneration results comparable to gold standard autografts, with uniformly distributed bone growth and limited ectopic bone formation (~28–35%), even at high BMP-2 doses. The BMP-2 coating not only promoted osteogenesis and angiogenesis but also ensured safety by effectively controlling ectopic bone formation and maintaining scaffold durability.
Abu-Shahba et al., 2022	**Superior Bone Regeneration and Remodeling with MVP Group**: The MVP group (muscle + vascularized periosteal flap) achieved the highest bone formation and lowest residual biomaterial volume among all groups. Periosteal flaps significantly enhanced vascularization, bone regeneration, and biomaterial remodeling. **Role of Periosteal Flaps in Bone Regeneration**: Vascularized periosteal flaps demonstrated predictable pro-vascularization and osteogenic potential. Bone regeneration was dependent on interaction with the mechanically stimulated local bony microenvironment post-transplantation, rather than on the periosteum’s intrinsic vascular supply during prefabrication. **Biomechanical and Histological Features**: Newly formed bone in the MVP group showed organized lamellar structures, integration with Sharpey’s fiber-like formations, and vascularized fibrovascular stroma, further boosting remodeling efficiency **Influence of Recipient Periosteum**: The preserved recipient site periosteum in the control group contributed significantly to bone regeneration, emphasizing the regenerative capacity of the periosteum.
Nokhbatolfoghahaei et al., 2022	**Enhanced Bone Formation with β-TCP Scaffolds**: β-TCP scaffolds demonstrated significantly higher rates of new bone formation compared to PCL/βTCP scaffolds, highlighting their potential as an effective solution for reconstructing critical-sized mandibular defects while simplifying surgical procedures and minimizing risks. **Role of rhBMP2 and MSCs**: Treatments with rhBMP2 and MSCs significantly promoted bone formation, vascularization, and osteogenesis, while reducing scaffold residues. Among all groups, the rhBMP2-treated pedicled β-TCP scaffold group achieved the highest bone regeneration rates, demonstrating the synergy of scaffold composition and biological enhancements. **Masseter Muscle as an In Vivo Bioreactor**: The masseter muscle served as an effective in vivo bioreactor, supporting scaffold prefabrication with minimal surgical complexity. This approach demonstrated promising outcomes for mandibular reconstruction, leveraging the muscle’s natural vascular and osteogenic properties.
Paré et al., 2022	**Bone Regeneration with Customized Constructs**: The calcium phosphate-based implant achieved successful regeneration of segmental mandibular defects (SMD) with full osseointegration and vascularization within 3 months. By 12 months, implants were entirely encased in lamellar bone, and healthy yellow marrow filled the remaining spaces, demonstrating long-term functional restoration. **Low Biodegradation Rate of BCP Scaffolds**: While biphasic calcium phosphate (BCP) scaffolds demonstrated excellent biocompatibility, their slow degradation left 75% of the initial ceramic volume intact after 12 months, limiting complete bone replacement and highlighting the need for improved scaffold materials with faster biodegradation. **Advanced Imaging with Deep Learning**: Deep learning algorithms significantly enhance segmentation accuracy for micro-CT analysis of bioceramic scaffolds, enabling a more precise assessment of bone formation, implant integration, and material performance.

## Data Availability

All data generated or analyzed during this study are included in the published article. Additional information can be provided by the corresponding author upon reasonable request.
